# Transcription Factor ChbZIP1 from Alkaliphilic Microalgae *Chlorella* sp. BLD Enhancing Alkaline Tolerance in Transgenic *Arabidopsis thaliana*

**DOI:** 10.3390/ijms22052387

**Published:** 2021-02-27

**Authors:** Dehui Qu, Pau-Loke Show, Xiaoling Miao

**Affiliations:** 1 State Key Laboratory of Microbial Metabolism, School of Life Sciences and Biotechnology, Shanghai Jiao Tong University, 800 Dongchuan Road, Shanghai 200240, China; qdhwd123@sjtu.edu.cn; 2 Joint International Research Laboratory of Metabolic & Developmental Sciences, Shanghai Jiao Tong University, Shanghai 200240, China; 3 Biomass Energy Research Center, Shanghai Jiao Tong University, Shanghai 200240, China; 4 Department of Chemical and Environmental Engineering, Faculty of Science and Engineering, University of Nottingham Malaysia Campus, Jalan Broga, Semenyih 43500, Selangor Darul Ehsan, Malaysia; PauLoke.Show@nottingham.edu.my

**Keywords:** alkaliphilic microalgae, ChbZIP1, *Arabidopsis*, detoxification pathway, alkaline tolerance

## Abstract

Saline-alkali soil has become an important environmental problem for crop productivity. One of the most effective approaches is to cultivate new stress-tolerant plants through genetic engineering. Through RNA-seq analysis and RT-PCR validation, a novel bZIP transcription factor ChbZIP1, which is significantly upregulated at alkali conditions, was obtained from alkaliphilic microalgae *Chlorella* sp. BLD. Overexpression of ChbZIP1 in *Saccharomyces cerevisiae* and *Arabidopsis* increased their alkali resistance, indicating ChbZIP1 may play important roles in alkali stress response. Through subcellular localization and transcriptional activation activity analyses, we found that ChbZIP1 is a nuclear-localized bZIP TF with transactivation activity to bind with the motif of G-box 2 (TGACGT). Functional analysis found that genes such as GPX1, DOX1, CAT2, and EMB, which contained G-box 2 and were associated with oxidative stress, were significantly upregulated in *Arabidopsis* with ChbZIP1 overexpression. The antioxidant ability was also enhanced in transgenic *Arabidopsis*. These results indicate that ChbZIP1 might mediate plant adaptation to alkali stress through the active oxygen detoxification pathway. Thus, ChbZIP1 may contribute to genetically improving plants’ tolerance to alkali stress.

## 1. Introduction

Microalgae have been considered as a promising feedstock for green renewable energy. Moreover, some microalgae also possess the ability to survive in extreme environments, such as alkaline conditions [1]. Recently, studies have reported that adversity tolerance of plants can be improved by genetic modification of heterologous genes [2,3]. Alkaliphilic microalgae is an ideal candidate resource for improving plant–alkaline tolerance [4]. Thus, understanding the microalgae alkali tolerance mechanisms and genetic engineering performance are the prerequisites for utilizing the alkaline environments for crop growth.

Transcription factors (TFs) play significant roles in response to plant defense and stress by activating or repressing the downstream target genes through specific binding with upstream cis-elements [5,6]. There are up to 320,370 TFs that have been identified in 165 plant species [7]. Based on the DNA-binding domains of TFs, they have been classified into 64 TF families in plant kingdoms [8]. The basic region-leucine zipper (bZIP) family is one of the most conserved and largest groups in the TF families. In plants, bZIPs play significant roles in metabolic processes, such as growth and development [9], signaling transduction [10,11], and various biotic/abiotic stresses [12,13].

In microalgae research, there has been increased attention on TF function studies. To date, a total of 147 TFs have been found in *Chlamydomonas reinhardtii*, involving eleven MYBs, seven bZIPs, five C2H2s, four bHLHs, two MADSs, and one WRKY [14]. One study found that bZIP TFs in *C. reinhardtii* play an important role in oil accumulation under salt stress [15]. In the diatom *Phaeodactylum tricornutum*, some bZIP TFs, like bZIP14 participate in the regulation of TCA biosynthesis pathways [16]. However, functions remain unclear for the majority of bZIP TFs in other microalgae, especially the extreme algae.

In this study, a novel bZIP TF ChbZIP1 of alkaliphilic microalgae *Chlorella* sp. BLD has been identified with transcriptome analysis, which was upregulated under alkali conditions. A functional study showed that ChbZIP1 could improve the alkali-resistant ability of yeast and *Arabidopsis*, and the antioxidant ability of *Arabidopsis* was enhanced with ChbZIP1 overexpression. This study facilitates a better understanding of the molecular mechanism of bZIP TF in responding alkali conditions, which provides an important foundation to further guide genetic engineering with microalgae in improving crop tolerance with extreme environments.

## 2. Results

### 2.1. Changes of Transcription Factors in the Alkali Stress

**Figure 1:**
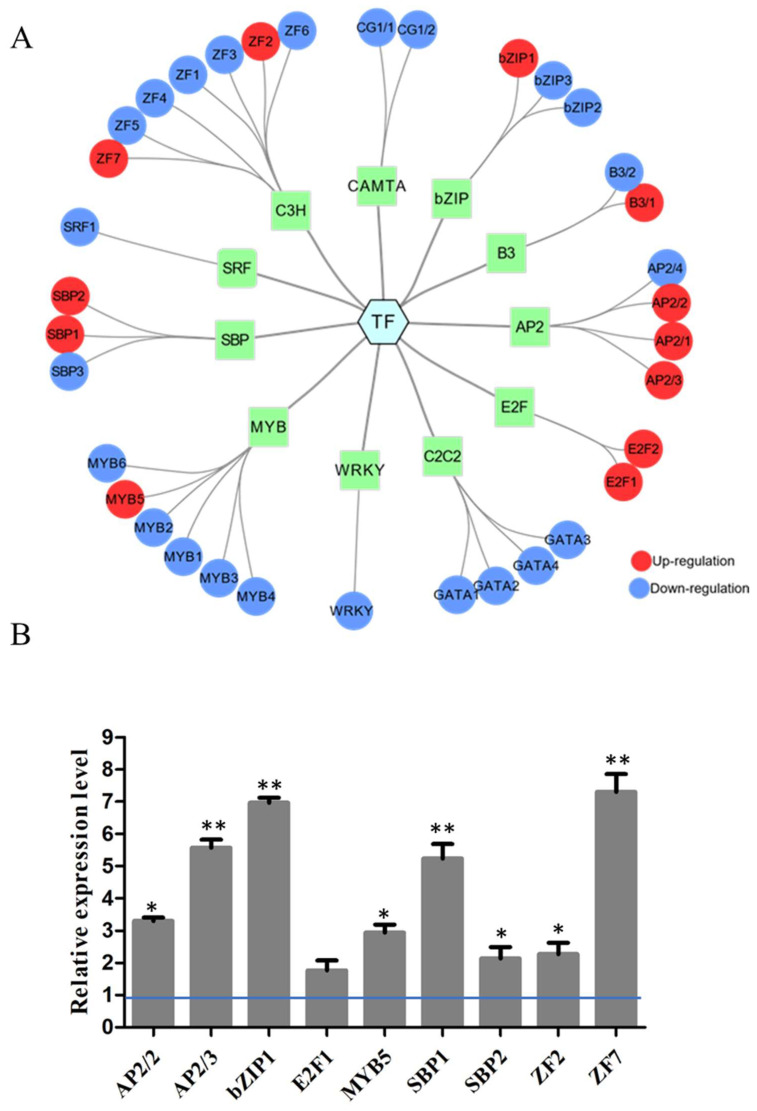
Analysis the expression of transcription factors of *Chlorella* sp. BLD under alkali conditions. (**A**) RNA-seq analysis the regulation of transcription factors (TFs) expression of *Chlorella* sp. BLD at pH 10 compared to pH 7.5. The results of the network analysis including TFs were analyzed and visualized using CYTOSCAPE. The red nodes indicate upregulation, and the blue nodes indicate downregulation. (**B**) Validation of the RNA-seq results of transcription factors (TFs) in upregulated by q-PCR. * indicates *p*-value < 0.05, ** indicates *p*-value < 0.01.

In our previous study, a new alkaliphilic microalgae *Chlorella* sp. BLD, was isolated and transcriptome analysis was performed to explore the mechanism of alkali resistance under pH 10. Through transcriptome analysis, a total of 338 TFs have been identified in *Chlorella* sp. BLD and can be classified into 20 families, which contain 48 MYBs, 42 C2H2, 35 C3H, 34 AP2, 30 C2C2, 22 bHLH, 21 bZIP, 16 WRKY and other TFs (Figure S1). Under alkaline conditions, there were 35 TFs including 12 upregulated and 23 downregulated TFs differentially expressed (Figure 1A). These TFs covered 4 AP2s, 3 bZIPs, 4 GATAs, 6 MYBs, 3 SBPs, 7 ZFs and 8 other TFs. To validate the expression level, 12 upregulated TFs were selected with q-PCR analysis. The results showed that the variation tendency of the TFs validated by q-PCR was similar to the data from RNA-seq (Figure 1B). Among these, four TFs (AP2/3, bZIP1, SBP1, ZF7) were significantly upregulated at alkali condition, which means these TFs may participate in the regulation of alkali stress.

### 2.2. Validation of the Alkali Resistance of TFs in S. cerevisiae

**Figure 2:**
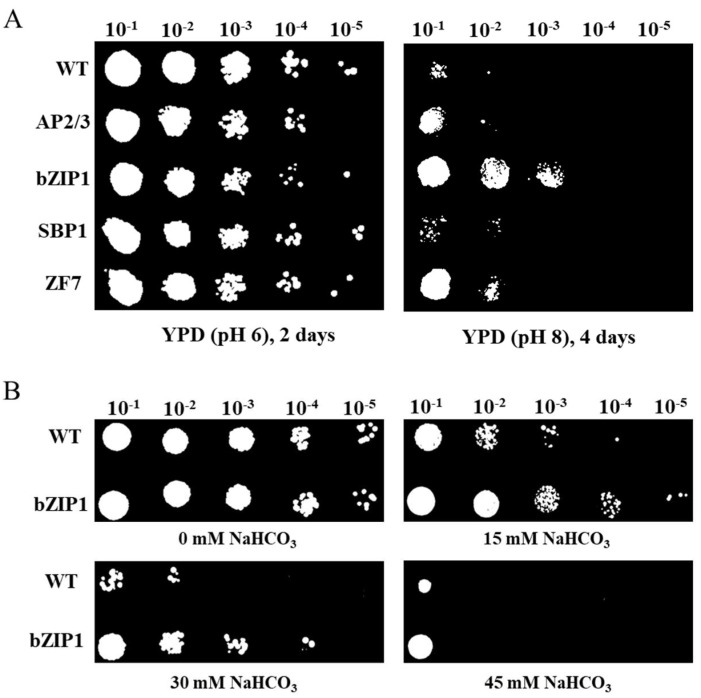
Transcription factor screening from *Chlorella* sp. BLD with alkali tolerance analysis in *S. cerevisiae.* (**A**) TFs (AP2/3, bZIP1, SBP1 and ZF7) were constructed to the pEGH-A vector and transformed into Y258 yeast. Serial dilutions were spotted onto pH 8 yeast YPD solid plates as alkali treatment, and on normal YPD (pH 6) solid plates as control. (**B**) Yeast cells with bZIP1 overexpression and WT were diluted and spotted onto the YPD solid medium supplemented with 0 mM, 15 mM, 30 mM, and 45 mM NaHCO_3_.

To understand the TFs’ function in alkali stress tolerance, the growth characteristics of wild type (WT) *S. cerevisiae* Y258 and strains overexpressing TFs (AP2/3, bZIP1, SBP1, and ZF7, respectively) at pH 8 YPD medium were surveyed after four days. As shown in Figure 2A, yeast with bZIP1 exhibited the best growth under alkaline conditions. More clones clearly appeared in bZIP1 transformed yeast than that of WT at dilution of 10^−2^ and 10^−3^ (Figure 2A). ZF7 and AP2/3 showed more colonies than WT at the dilution of 10^−2^ and 10^−1^, respectively. However, the transformants of SBP1 could not grow at pH 8 YPD medium with a dilution of 10^−1^, which similar to the growth tendency of WT. To further extensively explore the function of bZIP1 in alkaline condition, the growth of WT and bZIP1-overexpressed yeast cells were assayed under different concentrations of NaHCO_3_. The results showed that more colonies were found with bZIP1-overexpressed yeast than WT at 15 mM, 30 mM and 45 mM NaHCO_3_ with different dilutions (Figure. 2B). These results imply that the bZIP1, which is referred to as ChbZIP1 in the following, may play an important role in resisting alkali stress.

### 2.3. Characterization of the ChbZIP1 Sequence

**Figure 3:**
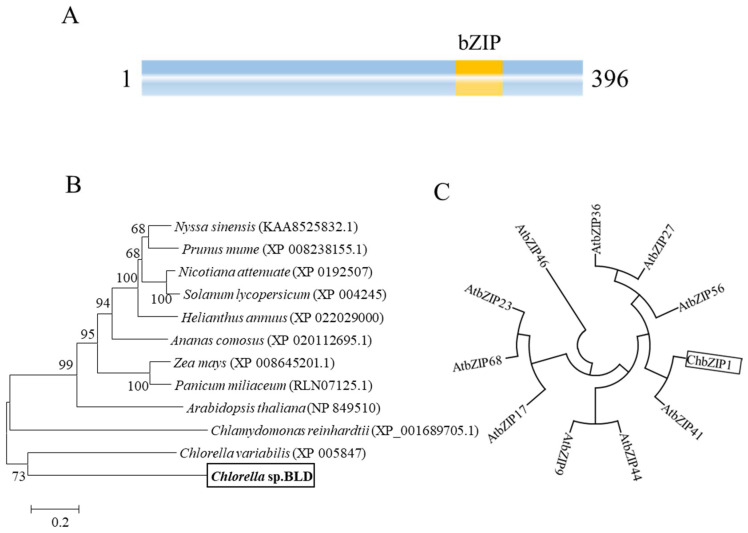
Phylogenetic and sequence analyses of ChbZIP1 from *Chlorella* sp. BLD. (**A**) Schematic representation of ChbZIP1 with basic leucine zipper (bZIP) domain. (**B**) Neighbor-joining phylogenetic relationships among ChbZIP1 from *Chlorella* sp. BLD and various bZIPs from other species. Bootstrap values were calculated at 1000 ties. *Chlorella* sp. BLD is indicated in boldface in box. (**C**) Neighbor-joining phylogenetic relationships among ChbZIP1 and ten groups of bZIP families in *Arabidopsis*. Box indicates ChbZIP1.

The ChbZIP1 TF belongs to the basic leucine zipper family, which is the third-largest TF family in *Chlorella* sp. [7]. Analysis of the ChbZIP1 sequence indicated that the ORF was 1188 nucleotides long and encoded a predicted protein of 396 amino acids. ChbZIP1 has a typical bZIP domain in the sequence (Figure 3A). Through neighbor-joining (NJ) tree analysis, ChbZIP1 showed a closer relationship with *Chlorella variabilis* (XP 005847) than other species. Meanwhile, the *Chlorella* sp. BLD first clustered with microalgae in the tree, then with plants (Figure 3B), which was in accordance with the expected taxonomy. To further explore the phylogenetic relationship of the ChbZIP1 in *Chlorella* sp. BLD, ten bZIP protein sequences from the *Arabidopsis* AtbZIP family were added into the tree and used as subgroup markers to classify the ChbZIP1. According to the circular NJ tree, ChbZIP1 was closer to AtbZIP41 of *Arabidopsis* (Figure 3C), which is classified into the G group.

### 2.4. Subcellular Localization and Transactivation Activity Analysis of ChbZIP1

**Figure 4:**
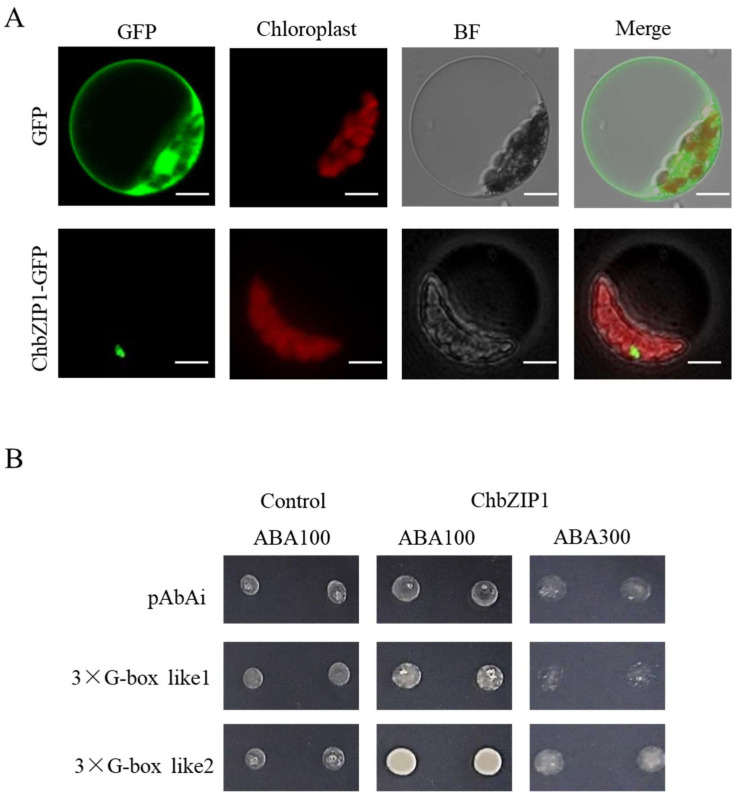
Subcellular localization and transactivation activity analyses of ChbZIP1. (**A**) Subcellular localization of ChbZIP1:GFP in *Arabidopsis* protoplasts. GFP empty vector is shown at the top. Bars = 10 µm. (**B**) ChbZIP1 binds the artificial, triple-repeated G-box 2 motifs in yeast cells at 100 and 300 ng/L concentration of aureobasidin A (AbA), whereas it fails to bind the G-box 1 and AbAi control vector.

Previous studies have reported that bZIP TFs were localized in nuclear [17], cytoplasmic [18], and endoplasmic reticulum [19]. Hence, to explore the subcellular localization of ChbZIP1 in plant cells, a ChbZIP1 sequence without the stop codon was constructed with the vector of pPZP211-GFP and transformed into *Arabidopsis* protoplasts. The result showed that the transformed protoplasts of ChbZIP1 exhibited GFP fluorescence in the nucleus (Figure 4A), suggesting that ChbZIP1 localized in the nucleus of *Arabidopsis* protoplasts.

In order to further explore the transcriptional activation activity of ChbZIP1, the motif of G-box 1 (CCACGT) and G-box 2 (TGACGT), which was plant bZIPs’ protein-binding motif [20], have been designed and verified by yeast one-hybrid (Y1H) experiments. To test the ChbZIP1 protein’s binding ability, triple repeated G-box 1 and G-box 2 motifs were designed and integrated into the genome of yeast cells. After introducing ChbZIP1 into the yeast strains, it was found that ChbZIP1 was preferred to bind the motif of G-box 2 (Figure 4B). Certainly, from the selection media SD/-L added with 100 ng/L and 300 ng/L Aureobasidin A (AbA), ChbZIP1 appears to have a higher affinity binding to the G-box 2 than to the G-box 1 motifs. The results of the yeast one-hybrid assay suggested that ChbZIP1 may participate in alkali responses by recognizing and interacting with the G-box2 motif.

### 2.5. Growth Analysis of ChbZIP1 Overexpression in Arabidopsis

To test the function of ChbZIP1 with alkali tolerance in plants, the cDNA of *ChbZIP1* was overexpressed in *Arabidopsis* under the control of promoter CaMV 35S. The band of *ChbZIP1* was detected with PCR screening in the six T1 (OX1-6) lines (Figure S2A). The above results confirmed that the *ChbZIP1* gene was successfully integrated into the genome of *Arabidopsis*. Furthermore, the mRNA expression level of *ChbZIP1* in T3 transgenic lines originated from the six identified T1 lines, indicating that *ChbZIP1* was successfully overexpressed in the *Arabidopsis* (Figure S2B). Three T3 lines, which highly expressed in *Arabidopsis*, named OX1, OX2 and OX4 were used for further functional validation.

**Figure 5:**
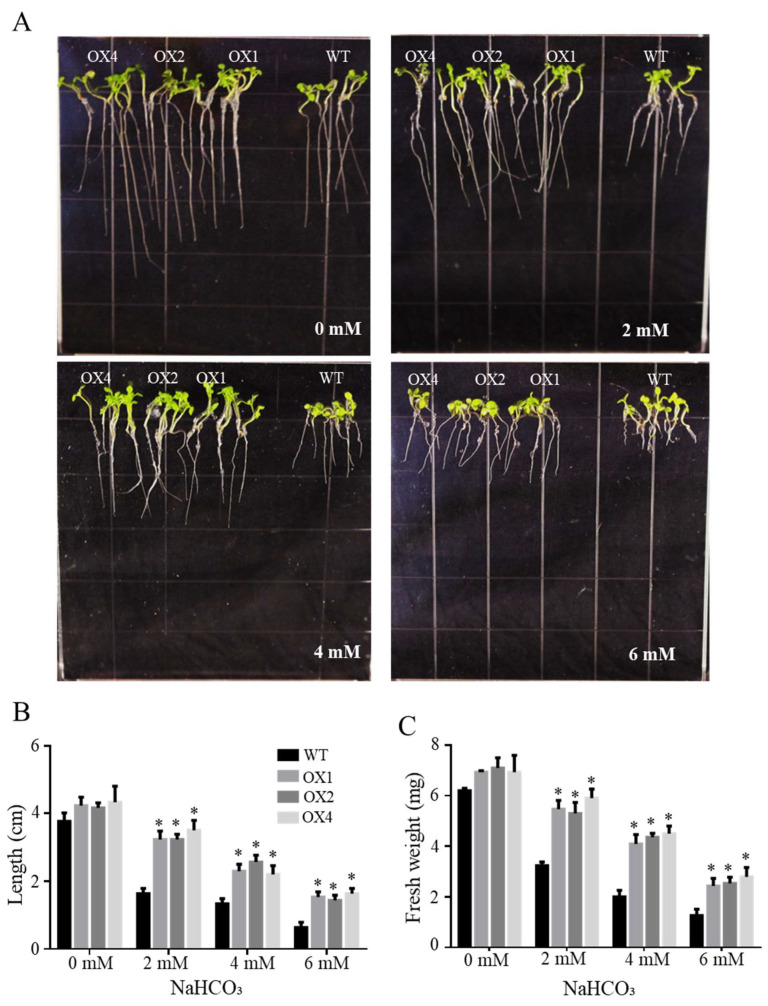
Effects of NaHCO_3_ stress on the growth of wild-type (WT) and ChbZIP1-overexpressed transgenic *Arabidopsis* plants. (**A**) Phenotypes of ChbZIP1 transgenic and WT lines subjected to 0, 2, 4, and 6 mM NaHCO_3_ treatment, respectively. (**B**) Root length changes in ChbZIP1 transgenic and WT lines. (**C**) Fresh weight changes in ChbZIP1 transgenic and WT lines. * indicates *p*-value < 0.05.

The NaHCO_3_ tolerance assay was used to detect the function of T3 lines (OX1, OX2 and OX4) of overexpressing ChbZIP1 in the *Arabidopsis*. The results showed that there were no significant changes between WT and transgenic lines (OX1, OX2 and OX4) in the growth on the 1/2 MS medium without NaHCO_3_ (Figure 5A). While under the treatment of NaHCO_3_, transgenic lines demonstrated the excellent alkali resistance ability compared with WT. Both the root growth (Figure 5A,B) and the entire seedlings fresh weight (Figure 5C) were significantly enhanced in transgenic lines compared with that in WT under 2 and 4 mM NaHCO_3_ treatment. Although the transgenic plants and WT all exhibited chlorosis at 6 mM NaHCO_3_ concentration, transgenic lines showed better adaptability with longer root length and higher fresh weight than WT.

### 2.6. The Antioxidant Ability in ChbZIP1-Overexpression Arabidopsis

**Figure 6:**
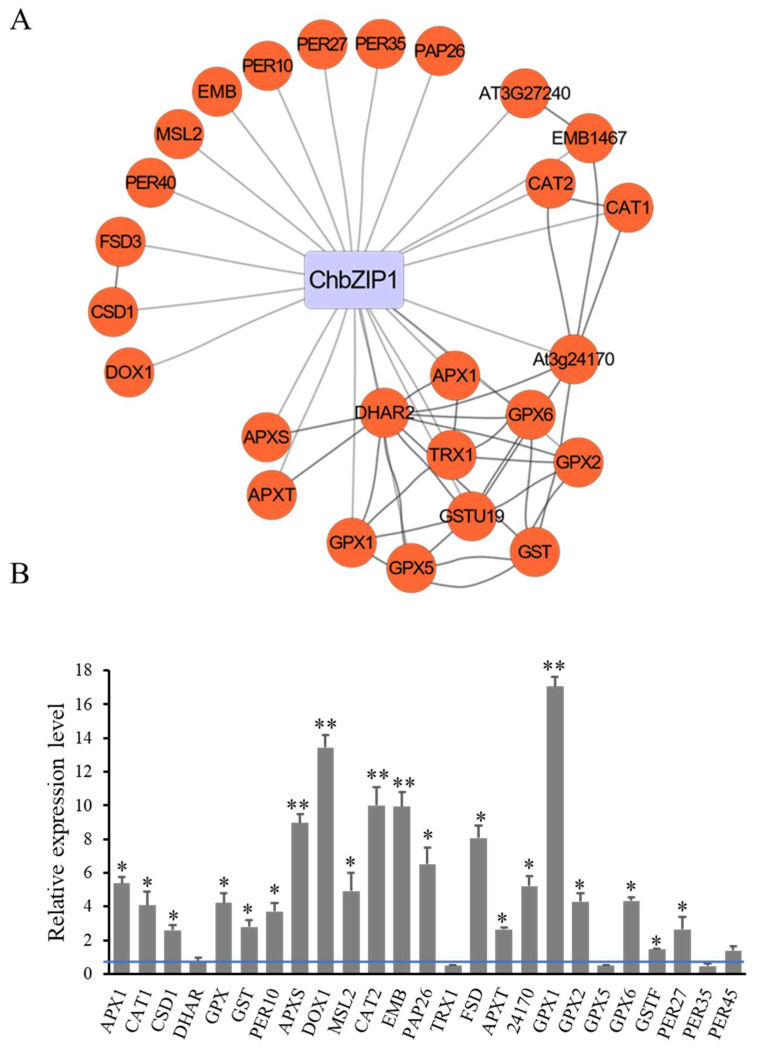
Screening and validation of antioxidant related genes which contained G-box 2 and may be regulated by ChbZIP1. (**A**) Antioxidant-related genes were extracted from the G-box 2 motifs’ binding database with STRING analysis and drawn by CYTOSCAPE. (**B**) Antioxidant related genes were detected compared to ChbZIP1 transgenic lines with WT lines seedlings exposed to 200 mM NaHCO_3_ treatment by q-PCR. * indicates *p*-value < 0.05, ** indicates *p*-value < 0.01.

Through yeast one-hybrid (Y1H) experiments, we found that ChbZIP1 showed high affinity binding to the G-box 2 (TGACGT). To further identify the function of ChbZIP1 in *Arabidopsis*, 1034 genes that contained G-box 2 motifs were obtained through Stress-Responsive Transcription Factor DataBase [21] analysis in *Arabidopsis*. After GO analysis, 25 oxidant detoxification related genes, which contained G-box 2 and may interact with bZIP TF, were isolated (Figure 6A). The expression level of these oxidant detoxification-related genes in ChbZIP1-overexpression and WT *Arabidopsis* were validated by qPCR (Figure 6B). The results showed that 84% (21/25) genes were highly upregulated (Figure 6B). Among the upregulated genes, *GPX1*, *DOX1*, *CAT2*, *EMB* and *APXS* have the highest expression level, followed by *FSD*, *PAP26*, *24170 (AT3G24170.1)*, *APX1*, *GPX2*, and *CAT1*. These results indicated that ChbZIP1 may enhance the antioxidant ability of *Arabidopsis* by regulating the redox-related genes, thus improving the alkali tolerance in plants.

**Figure 7:**
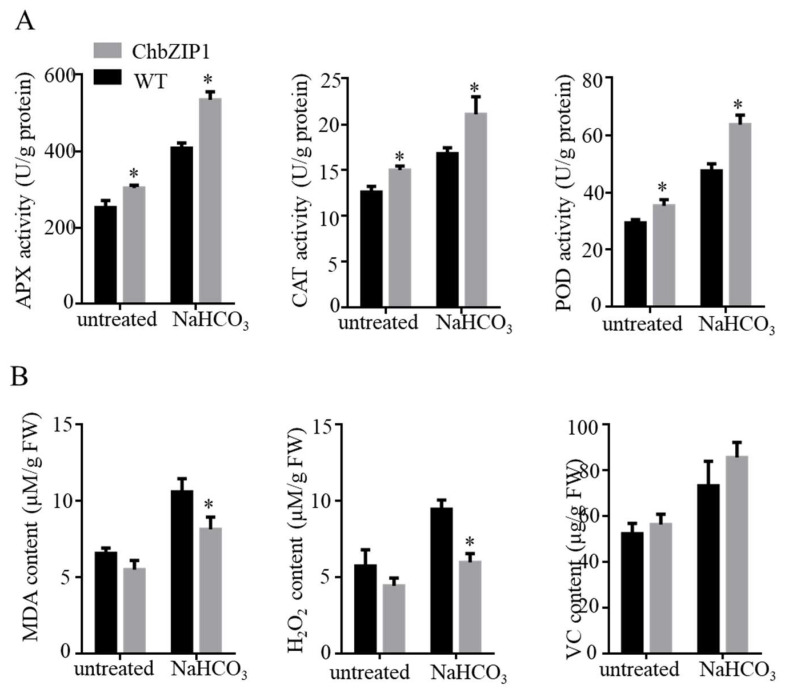
Effects of NaHCO_3_ stress on the antioxidant ability in wild-type (WT) and ChbZIP1 overexpressed transgenic *Arabidopsis* plants. (**A**) Measure of the enzymatic activities of APX, CAT, and POD in ChbZIP1 transgenic and WT seedlings compared to NaHCO_3_ stress and control. (**B**) Measure of the content of MDA, H_2_O_2_, and Vc in ChbZIP1 transgenic and WT seedlings compared to NaHCO_3_ stress and control. * indicates *p*-value < 0.05.

To verify the above inference, the activities of ROS scavenging enzymes like ascorbate peroxidase (APX), catalase (CAT), and peroxidase (POD) and ROS accumulation were measured. In this study, APX, CAT, and POD all exhibited higher enzyme activities in the ChbZIP1 transgenic line compared to WT (Figure 7A). The increase of antioxidant enzyme activity reduced the ROS content and thus improved the tolerance of plants to stressed environments [22]. The results of ROS accumulation are consistent with the activities of ROS scavenging enzymes. The content of malondialdehyde (MDA) and hydrogen peroxide (H_2_O_2_) showed no significant differences in ChbZIP1 transgenic plants compared with those in an untreated state in WT (Figure 7B). While under the NaHCO_3_ stress exposure, the content of MDA and H_2_O_2_ in the transgenic strains was dramatically decreased compared with that in WT (Figure 7B). However, there was no significant change in the Vitamin C (VC) content between WT and transgenic lines.

## 3. Discussion

In recent years, the area of saline-alkali soil has gradually increased, which resulted in severe losses in agricultural production. Previously, we had isolated an alkaliphilic microalgae *Chlorella* sp. BLD, which can grow well in extreme alkali conditions at pH > 10. In transcriptome analysis, bZIP transcription factor ChbZIP1 was highly up regulated at pH 10 compared to normal conditions (Figure 1). The functional study also found that overexpression of ChbZIP1 in *S. cerevisiae* and *Arabidopsis* could improve their alkali resistance. Meanwhile, ChbZIP1 could also enhance the antioxidant ability of *Arabidopsis* with related genes and ROS-related enzymes. Together, these findings not only elucidate the unprecedented role of ChbZIP1 in regulating alkali resistance but also provide a new direction for plant genetic engineering.

### 3.1. ChbZIP1 May Play Important Roles in Resisting Alkali Stress

The ChbZIP1 gene was isolated from the cDNA of *Chlorella* sp. BLD, and structure analysis showed a typical bZIP domain in its sequence (Figure 3A). Evolutionary relationship analysis with *Arabidopsis* found that ChbZIP1 was classified into group G (Figure 3C). The homologous proteins of ChbZIP1 in *Arabidopsis* were AtbZIP16, AtbZIP41 (GBF1), AtbZIP54 (GBF2), and AtbZIP55 (GBF3) [20]. It was reported that the group G *AtbZIP* genes from *Arabidopsis* were mainly linked to light signal transduction, regulation of gibberellic acid (GA), and abscisic acid (ABA) response [23,24]. In our study, ChbZIP1 overexpressing in *S. cerevisiae* and *Arabidopsis* significantly improved their tolerance to alkali stresses (Figure 2 and Figure 4). Studies reported that bZIP TF could improve the survival of crop plants in alkaline conditions. A bZIP TF GsbZIP67, which was isolated from Glycine soja, overexpressed in alfalfa promoted plant growth under bicarbonate alkaline stress, as evidenced by longer roots and shoots [17]. DuanMu et al. also found that the bZIP TFs family was significantly induced in the alkaline stress responses of wild soybean roots through transcriptome analysis [25]. All these results indicated that bZIP TFs play an important role in response to the alkaline condition.

### 3.2. ChbZIP1 Is A Nuclear-Localized bZIP TF with Transactivation Activity

Members of the bZIP TF family participate in the regulation of various biological processes such as environmental responses, plant growth, and development. One study reported that bZIP TF can bind to elements that contains a functional ACGT core, like G-box, C-box, and A-box [26]. Our results showed that ChbZIP1 is a nuclear-localized bZIP TF with transactivation activity through subcellular localization and transcriptional activation activity analyses (Figure 4). ChbZIP1 preferentially binds with the G-box2 motif (TGACGT). Proteins (AtGBF1, AtGBF2, and AtGBF3) belonging to the same G-group as ChbZIP1 have been shown to bind with the G-box element, which mediates expression in response to different stimuli [27]. Thus, there is an extremely high probability that ChbZIP1 plays an important role in response to alkaline stress by binding with the G-box2 motif.

### 3.3. ChbZIP1 May Enhance the Antioxidant Ability through Regulating Oxidant Detoxification-Related Genes with G-Box 2 Motif

Genes related to oxidation reduction play significant roles in stress response, because they can scavenge the ROS and maintain the redox balance under stress [28]. In this study, we have found 25 oxidant detoxification related genes that contain G-box 2 and may interact with bZIP TF (Figure 6A). Through qPCR analysis, it is found that 84% (21/25) of genes related to oxidant detoxification are highly upregulated in ChbZIP1-overexpression *Arabidopsis* (Figure 6B). These results indicate that ChbZIP1 might enhance the antioxidant ability of *Arabidopsis* by regulating the redox-related genes, thus improving the alkali tolerance in plants. The activities of ROS scavenging enzymes (APX, CAT, and POD) and ROS accumulation were measured and verified this inference (Figure 7).

Superoxide dismutase (SOD), ascorbate peroxidase (APX), glutathione peroxidase (GPX), and catalase (CAT) are key antioxidative enzymes, which could counteract the damage of ROS. For example, the plant GPX family, which is upregulated by ChbZIP1 in our study, could detoxify H_2_O_2_ and organic hydroperoxides [29]. Besides that, the present study also finds that GPX is involved in regulating the cellular redox homeostasis by maintaining the thiol/disulfide or NADPH/NADP^+^ balance [30]. The APX as a component of the ascorbate-glutathione cycle could maintain in the appropriate level of H_2_O_2_ in organelles and cytosol [31]. Overexpression APX in potato and *Arabidopsis* exhibits high tolerance to abiotic stress, such as oxidative stress, chilling, salt and methyl viologen (MV) stress [32,33]. The CAT family, which contains three genes (CAT1-3) in most plant species, scavenges H_2_O_2_ in millimolar concentrations, especially in peroxisomes [34]. Some reports have implied that CAT1 plays an important role in signaling related to irradiance and abscisic acid (ABA) [35,36].

**Figure 8:**
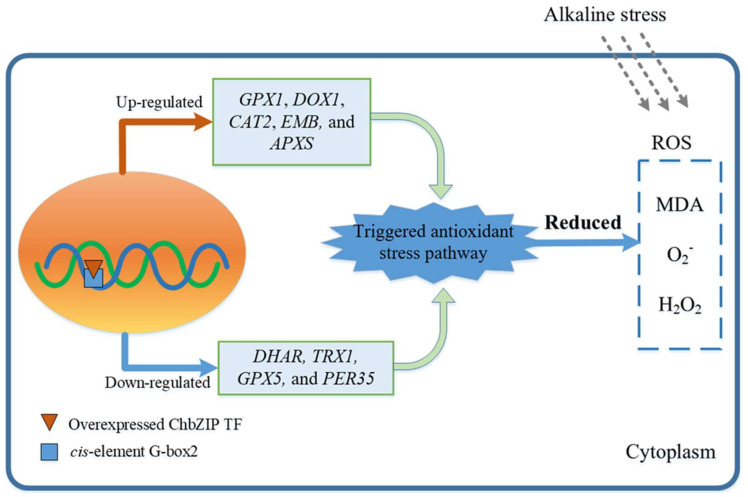
The proposed alkaline tolerance mechanism in *Arabidopsis* with overexpressed ChbZIP1 TF.

These results indicate that ChbZIP1 could regulate the oxidant detoxification-related genes with the G-box 2 motif, further enhance the activities of the enzymatic antioxidants, and reduce oxidative stress damage in the transgenic *Arabidopsis* lines (Figure 8). Thus, ChbZIP1 could be a potential gene for improving plant tolerance in alkali stress.

## 4. Methods and Materials

### 4.1. Microalgae Cultivation

The algae *Chlorella* sp. BLD used in this study was presented by Ghopur Mijit (Xinjiang University) and cultivated with the BG-11 medium. The pH 7.5 (maintained with 25 mM Hepes) and pH 10 (maintained with 25 mM Caps) of the BG-11 medium was adjusted by NaOH. The temperature of cultivation was 25 ± 1 °C and light intensity was 160 μmoL photons m ^−2^ s ^−1^ in 1 L Erlenmeyer flask.

### 4.2. Validating the Expression of Genes with qPCR

Quantitative real-time PCR (qPCR) was used to verify the genes expression. RT-qPCR was measured by SYBR SuperReal PreMix Plus (Tiangen, Beijing, China) in Real-Time PCR Detection System (Bio-Rad, Hercules, CA, USA). The housekeeping gene β-tubulin and UBQ10 (AT4g05320) were used as an internal standard for *Chlorella* sp. BLD and *Arabidopsis*, respectively. Primers used in this study for qPCR analysis are shown in Table S1. The relative expression levels were calculated using the 2^−∆∆*C*t^ method [37].

### 4.3. Analysis of Resistance to Alkali Stress in S. cerevisiae with TFs Overexpression

To detect the related transcription genes’ response to alkali conditions, four highly-expressed genes were amplified and cloned into vector pEGH-A (yeast expression vector) with the Gateway cloning system. Using the LiAc method, the success clones were transformed into the *S. cerevisiae* strain Y258. To select the successful expressed plasmids, the medium of SD-Ura was used for yeast strains cultivation. The TFs’ overexpressed yeast cells and WT were adjusted from OD_600_ to 0.1 for alkali tolerance analysis. Then preparation for a serial of dilutions with ten-fold and was spotted on a solid YPD medium (pH 6 and pH 8) with 5 μL aliquots of each dilution for incubation at 30 °C for 2 days and 4 days, respectively.

### 4.4. Construction of Phylogenetic Trees

The sequence of ChbZIP1 was screened from the RNA-seq results. The homologous sequences of other species were searched using the program NCBI-pBLAST. For tree construction, the sequences that most closely matched the sequence of ChbZIP1 were obtained by BLAST from the GenBank database. Clustal X was used to align the similar sequences. The tree was constructed using the method of neighbor-joining with the MEGA5.0 software [38].

### 4.5. Subcellular Localization Analysis of ChbZIP1 in Arabidopsis Protoplasts

The open reading frame (ORF) of the *ChbZIP1* sequence without the stop codon was amplified and constructed into the pPZP211-GFP vector with the *Kpn* I and *Xbal* I sites. Then, the recombinant plasmid was transiently expressed in *Arabidopsis* protoplasts by PEG-mediated transformation according to the method of Yoo et al. [39]. The *Arabidopsis* protoplasts were then incubated at 23 °C for 16 h with protein expression and the fluorescence of GFP and autofluorescence of chlorophyll were observed with a confocal laser-scanning microscope at 488 nm and 561 nm, respectively.

### 4.6. Yeast One-Hybrid Assays

The TF–DNA interaction experiments were performed using the Gold Yeast One-Hybrid system [40]. G-box motif 1 and G-box motif 2 with triple tandem copies were inserted into the reporter plasmid pAbAi. The bait constructs were linearized and then integrated into the genome of the Y1H Gold yeast stain. Transformants were selected on a synthetic defined SD/-Ura agar medium plate and cultured at 30 °C for 2 days. The *ChbZIP1* gene was constructed to the yeast activation vector of pGADT7 as prey. The prey construct was then transformed to the yeast cells, which contained the bait constructs. SD/-Leu medium with different concentrations of aureobasidin A (AbA) were used to culture the positively transformed clones.

### 4.7. Functional Analysis of ChbZIP1 Overexpression in Arabidopsis

The PCR product of *ChbZIP1* was cloned into the expression vector pBI121. For *Arabidopsis* transformation, the *pBI121::ChbZIP1* was first transformed into *Agrobacterium tumefaciens* GV3101 by chemical method. Then, the floral dip method was used to transform the *Arabidopsis* plants [41]. Half-strength Murashigeand Skoog (1/2 MS) medium was used for selecting the *Arabidopsis* transgenic seeds with 50 mg L^−1^ kanamycin. To further explore the function of ChbZIP1 with the resistance of alkali stress, the transgenic T3 generation and WT seeds were plated on the 1/2 MS medium containing 0, 2, 4, and 6 mmol L^−1^ NaHCO_3_ (concentration gradient was set up according to the preliminary experiment and previous studies [42]) for germination. After 10 days, the length of roots was measured. In addition, when the seedlings grew to the four-leaf stage, they were transplanted to the soil in the greenhouse at 23 °C under 12 h light/ 12 h dark cycles. After 30 days, the transgenic and wild type plants were treated with 200 mM NaHCO_3_ for one week. The content of MDA, H_2_O_2_ [43] and Vc [44], were measured. APX, CAT, and POD activities were measured as described in a previous study [45].

### 4.8. Statistical Analysis

All data were presented as the means ± standard errors of at least three biological replicate samples (GraphPad Prism 7.03). One-way ANOVA followed by a T test were done for statistically significant results with *p*-value < 0.05 (IBM SPSS 24).

## Supplementary Materials

The following are available online at https://www.mdpi.com/1422-0067/22/5/2387/s1, Figure S1: Transcription factors were founded in Chlorella sp. BLD through transcriptome analysis, Figure S2: Overexpressed ChbZIP1 in Arabidopsis. A. The band of ChbZIP1 was detected with PCR screening in the six T1 (OX1-6) lines. B. The mRNA expression level of ChbZIP1 in T3 transgenic lines, Table S1: Primers used in this study.
