# Supplementary material for: Transcription Factor ChbZIP1 from Alkaliphilic Microalgae Chlorella sp. BLD Enhancing Alkaline Tolerance in Transgenic Arabidopsis thaliana
Source: Int J Mol Sci. 2021 Feb 27;22(5):2387. doi: 10.3390/ijms22052387 (PMC7957498; doi:10.3390/ijms22052387)
Supplement: Supplementary file 1 [file ijms-22-02387-s001.pdf]

Table S1 Primers used in this study.

Name	Sequence 5'-3'
β-tubulin-F	CAGTACCGTGCCCTGACA
β-tubulin-R	ATAGACATGCGGCCCCTG
AP2/2-F	GAGCCCCTTGGAGTCGAG
AP2/3-R	TGACGGGGTTGGATCGAC
AP2/3-F	GCGTTCAGGCCCCAAATC
AP2/3-R	GGGCCCTCTGAACCGAAT
bZIP1-F	GCGAAAGAAACGGAGGGC
bZIP1-R	GCACCGGTGTTGGGAAAG
E2F1-F	ACGAGTTCCCACGAGCTG
E2F1-R	TCCCGAAGCTTTGAGTCG
MYB5-F	CCCATGGGTTCGAGGTGT
MYB5-R	AAGGAGAAACGGACGCGA
SBP1-F	TCCACGCCACGCTATCTC
SBP1-R	TTGATTGGCCCCATCGGT
SBP2-F	TCATATTTGCCCGGCCCA
SBP2-R	CGAAGTGGAGGAAGGCGA
ZF2-F	CTGCCATGCTTCAACACG
ZF2-R	GACGTGGAACACCCAAGG
ZF7-F	CGGATGGGGAGCTTGGAA
ZF7-R	TACTTGCGAGACCCAGGC
At-UBQ10-F	AAACCCTAACGGGAAAGACG
At-UBQ11-R	GAGTTCTGCCATCCTCCAAC
At-APX1-F	GCATGGACATCAAACCCTCT
At-APX1-R	AGCAAACCCAAGCTCAGAAA
At-CSD1-F	ACATTTCAACCCCGATGGTA
At-CSD1-R	CCAGTAGCCAGGCTGAGTTC
At-CAT1-F	ACTCCAGACCCGGATCTTCT
At-CAT1-R	CACGATTTCCAGAGCAGACA
At-PER10-F	CGCTTCCTCAGCACTTCTCT
At-PER10	CTCGCTGTACGACTTCACCA
At-GPX-F	ACGAGGAACCGGGAACTAAT
At-GPX-R	GAATGTCATCGCCGAAGATT
At-DHAR-F	TAATGACGGATCCGAGAAGG
At-DHAR-R	AAGCTCTCAGGGACAGACCA
At-APXT-F	TGGAGAAGCAGGAGGACAGT
At-APXT-R	GCAGCCACATCTTCAGCATA
At-GST-F	GGCGATGACTTTGGCTATGT
At-GST-R	CTTCTCAGGATCAGGCAAGG
At-GPX1-F	TCCTCAGCAACCTTTCTCGT
At-GPX1-R	CATCCTTCCCATCAATGTCC
At-GPX2-F	CAAATGTGGTCTGACGGATG
At-GPX2-R	GATGGGGAATTCAGCTTTGA
At-PER27-F	GGTTGCACTTGAAGGACCAT
At-PER27-R	GTGTGACCACCCGAGAGAAT
At-DOX1-F	GGAATGAAGAAACCGCAAAA

At-DOX1-R	CCTTGCGACCAATCAAATCT
At-24170-F	TTTTGCGAACACTGCTTTTG
At-24170-R	AGCCTGAGGTGAAGACCAGA
At-PER35-F	AATTCAACGGCATCAACTCC
At-PER35-R	TTTGAGGCCCAAGCATTAAC
At-TRX1-F	TTGAGACATGGAACGAGCAG
At-TRX1-R	CCTGTATCGCCCAATCACTT
At-EMB2360-F	AAGGCAAAAGAAGGTGCTGA
At-EMB2360-R	ACATGCGAAAATCCCTCAAC
At-MSD2-F	ATGGAACCGTGTCTCGAATC
At-MSD2-R	AGTTTGACGACGGAGGAGTG
At-GPX5-F	GTTTTCCAAAAGGTGCGTGT
At-GPX5-R	TTCTTGTGCAAGGGCTTTCT
At-GSTF-F	CAAGGAACCAACCTTCTCCA
At-GSTF-R	GCCTCCTCTTCTGCAACAAC
At-APXS-F	GGTCCTGAGGATTGTCCAGA
At-APXS-R	CTCCAGGTCCTTCTTTCGTG
At-GPX6-F	ATTCGATTCTGCGAAACCAC
At-GPX6-R	CAAGCCACATTGAGAAGCAA
At-PER45-F	TAGGGAGGAGAGATGGCAGA
At-PER45-R	TCGGGTCGATACGTGTTGTA
At-CAT2-F	CGAGGTATGACCAGGTTCGT
At-CAT2-R	AGGGCATCAATCCATCTCTG
At-FSD3-F	GGATGTGTGGGAGCACTCTT
At-FSD3-R	GATTGGGATGTTGGGTTCAC
At-PAP26-F	ATAGCTGGGGTCGTTTTGTG
At-PAP26-R	CAAAAGGCGAATAGCTGGAG

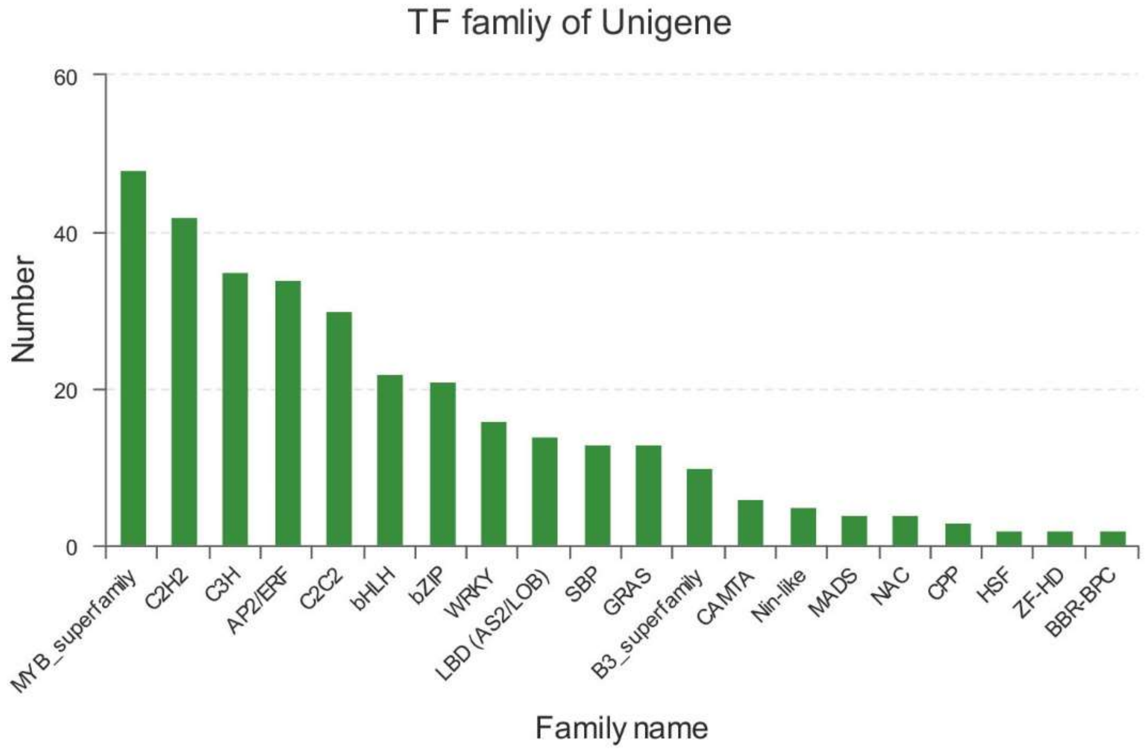

Figure S1. Transcription factors were founded in *Chlorella* sp. BLD through transcriptome analysis.

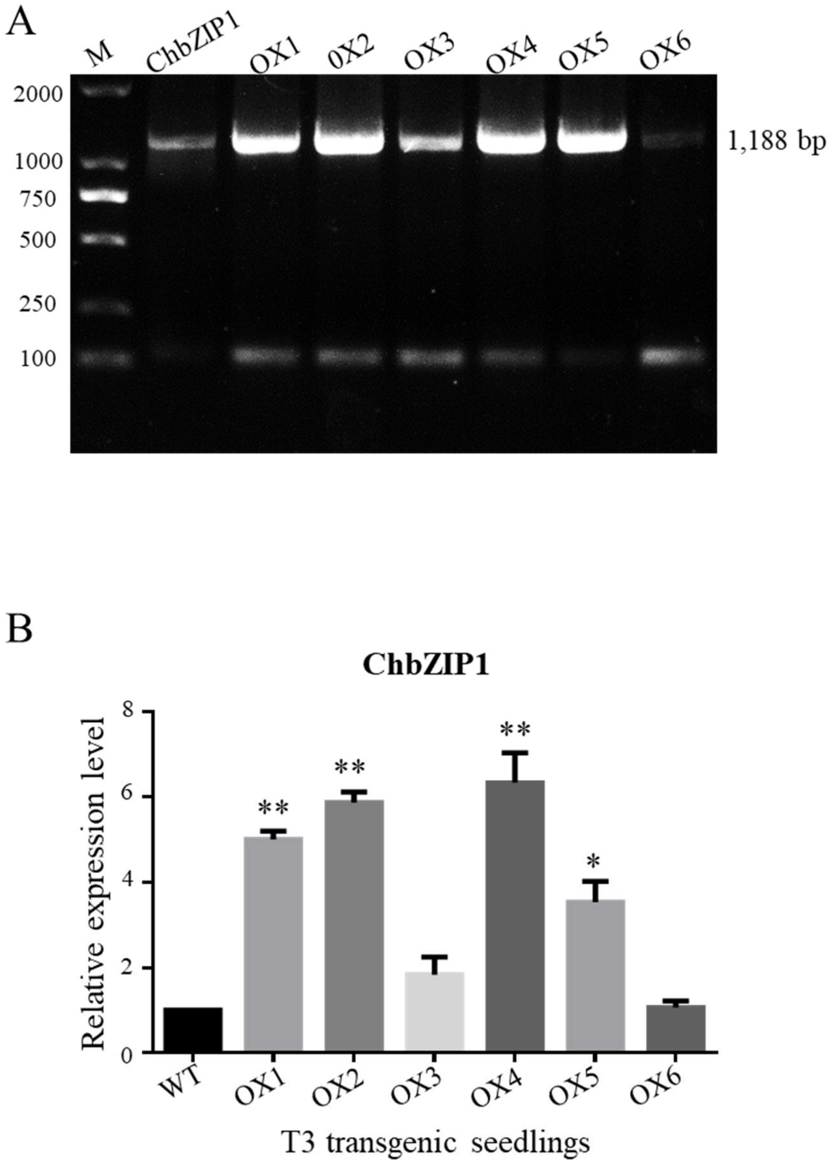

Figure S2. Overexpressed ChbZIP1 in *Arabidopsis*. A. The band of ChbZIP1 was detected with PCR screening in the six T1 (OX1-6) lines. B. The mRNA expression level of ChbZIP1 in T3 transgenic lines.
